# Inter-individual differences in laboratory rats as revealed by three behavioural tasks

**DOI:** 10.1038/s41598-022-13288-w

**Published:** 2022-06-07

**Authors:** Veronika Rudolfová, Tomáš Petrásek, Eliška Antošová, Daniel Frynta, Eva Landová, Karel Valeš, Tereza Nekovářová

**Affiliations:** 1 Department of Zoology, Faculty of Science, Charles University, Prague, Czech Republic; 2 Department of Physiology, Faculty of Science, Charles University, Prague, Czech Republic; 3 Institute of Physiology, Czech Academy of Sciences, Prague, Czech Republic; 4 National Institute of Mental Health, Klecany, Czech Republic; 5 Department of Ethology and Companion Animal Science, Faculty of Agrobiology, Food and Natural Resources, Czech University of Life Sciences, Prague, Czech Republic

**Keywords:** Zoology, Animal behaviour

## Abstract

Stable inter-individual differences in behaviour and personality have been studied for several decades now. The aim of this study was to test the repeatability of behaviour of the Long Evans strain of laboratory rats in order to assess their inter-individual differences. Male laboratory rats (n = 36) were tested in a series of tasks (Open field test, Elevated plus maze test, and modified T-maze test) repeated over time to assess their personality traits. To evaluate the temporal stability of the behaviour, we calculated repeatability estimates of the examined traits. We also checked for a link in behavioural traits across these experiments, which would suggest the existence of a behavioural syndrome. We found stable inter-individual differences in behaviour. Interestingly, no link emerged between the tasks we studied and therefore we did not find support for a behavioural syndrome. The lack of behavioural correlations between these experiments suggests that the results derived from these tasks should be interpreted carefully, as these experiments may measure various behavioural axes. Moreover, the animals habituate to the apparatus. Consequently, behaviour in the Open field test and Elevated plus maze test is not fully consistent and repeatable across subsequent trials.

## Introduction

Different individuals of the same species do not behave the same as has already been reported for many species (see^1–4^). Particularly songbirds and rats have been studied the most in this regard^5^. Even though inter-individual behavioural differences are more pronounced in wild and wild-derived animals^1,6,7^, some differences may also be found between laboratory animals^1^. In laboratory animals, however, the focus is on the differences in personality traits between various strains^8^. These are crucial for designing pharmacological experiments, where it is customary to select a single strain based on its behaviour in various experimental procedures. Different individuals of the same strain, on the other hand, are considered rather identical and in some research areas, it is customary to conduct experiments on fewer than ten individuals (e.g.,^9–11^), because very limited variability of behaviour is expected. Nevertheless, in this paper we would like to point out that there are also inter-individual differences within a single strain which are not negligible. The interpretation of results obtained from studies with a low number of individuals should therefore be more cautious.

### Personality

Inter-individual behavioural differences, consistent in time and throughout contexts, are of special research interest. Although terms referring to this variability may differ depending on the field of the study, “personality” is the term most widely used in the behavioural sciences, with synonyms such as temperament, behavioural profile, or others^4,12^. In human and primate studies, personality is described by a 5-axis paradigm (with each axis representing a continuum of behaviours) derived from The Big Five and NEO personality questionnaires^13–15^, while fewer axes are used in most of the animal taxa, see for example^2–4^. Individual scores on these axes should comprehensively describe animal behaviour in any experimental or real-life situation. Nevertheless, most studies do not test the full spectrum of animal behaviour, but rather focus on traits corresponding to only one or two axes (see a meta-analysis^6^ or a review^2^; see also Table S1 in Supplementary materials for experiments commonly used for extraction of personality axes). Correlation of any two axes is referred to as a behavioural syndrome, which is manifested when the behaviour of an animal in one context is dependent on its behaviour in another situation, possibly reflecting limited behavioural plasticity^16,17^.

### Behavioural consistency

Although it is assumed that inter-individual differences in behaviour are relatively stable in time, it does not mean the behaviour of an individual animal cannot change^6,18^. Nevertheless, relative differences among individuals should remain consistently stable throughout different contexts^19–21^.

When testing for behavioural consistency, two types of repeatability estimates can be distinguished. The first one, agreement repeatability (R_A_), expresses the reproducibility of the absolute scores the of behaviour of the animals tested, therefore it does not account for changes of behaviour in time^6,22,23^. In general, repeatability estimates vary from 0 (no repeatability) to 1 (maximum repeatability), with the average R_A_ estimate of personality traits of 0.37^1^. If there is a shift in behaviour between experimental trials (for example due to habituation), the agreement repeatability estimate would underestimate the “real” repeatability of behaviour^18^. In contrast, the second type of repeatability estimate, consistency repeatability (R_C_), takes changes in time (e.g., habituation) into account and therefore enables researchers to assess the relative scores of individual animals (e.g., by comparing the ranks of individual animals in several tasks) concerning a behavioural trait^6,18^.

### Methods used for personality testing

Personality is defined as stable inter-individual differences in behaviour. There are, therefore, a myriad of possibilities to test for such differences and diverse methods have become commonly used when testing various species (for details on birds, fish, rodents, or wild animals see^5^). In rodents, three axes are studied most often—activity, exploration, and boldness^6^. Activity may be measured in any experimental setup and it is quite easily assessed by simply measuring the path the animal travels (for references see Table S1). The exploration—avoidance axis represents the reaction to a new (not necessarily risky) situation^4^ and in rodents, it is usually assessed in experiments such as Open field test (empty arena), Hole board test (arena with holes in the ground), or in more complex novel environment designs (for references see^6^ or Table S1). The third most commonly tested axis is boldness, which corresponds to the reaction of the individual to a risky situation^4^. In rodents, this trait is usually assessed by the Elevated plus maze (elevated X-shaped apparatus with two open and two closed arms), Novel object test, or Startle test (for references see Table S1). The boundaries between these three dimensions (or axes) are, however, rather blurred as can be seen by the disagreement on the interpretations of the behavioural traits recorded in these procedures (e.g.,^24–28^). It is, for example, quite intriguing to decide whether behaviour in a brightly lit Open field test represents exploration or boldness and whether, on the other hand, behaviour in a dimly lit Elevated plus maze might represent exploration, rather than boldness. Moreover, in studies that do not consider personality, these procedures are used to measure the anxiety of the animals (e.g.,^29,30^). But how does anxiety translate into personality dimensions? Is it analogous to boldness or to the exploration-avoidance axis? In addition, activity is an inherent and inseparable feature of most of the behaviours recorded in these procedures, and therefore it is rather interesting to differentiate between the various personality axes. Furthermore, subsequent exposures to the same experiment always present a challenge in the interpretation of the behaviour (e.g.,^31,32^). Especially when considering personality and deciding whether the situation is new and potentially risky to the animal or whether it remembers the apparatus and therefore, the experiment is not a dangerous new threat any more.

In this study we used three standard experimental procedures—Open field test, Elevated plus maze test, and a modified version of the T-maze test. The Open field test is an empty apparatus with walls which prevent the animal from escaping it. At first, this apparatus was used to test the emotional reactivity of animals, nowadays it is mostly used to assess activity, anxiety, or exploration (reviewed in^29^, for more details see the Methods section). The Elevated plus maze test is an elevated X-shaped apparatus with two closed and two open arms (the same arms are located opposite each other, for more details see the Methods section). This experiment is used to test the exploration-avoidance axis and risk assessment (reviewed in^30^). The T-maze (or its alternative, the Y-maze) is used to study spontaneous alternation^33^, anxiety or exploration-avoidance axis^34^, and working memory^35^ (for more details see the Methods section). Even though these procedures test equivalent behavioural traits, studies adopting a multivariate analytical approach often show that the behaviours measured in these procedures create separate axes and therefore should not be interpreted as corresponding, e.g.,^34,36,37^.

The aim of this study was to examine inter-individual differences in behaviour in the Long Evans strain of laboratory rats. To this end, we utilized a battery of standard experimental procedures consisting of the Open field test, Elevated plus maze test^38–40^, and a modified version of the T-maze test^34^. These procedures are usually interpreted as tests of activity (Open field test), exploration (Open field test, Elevated plus maze test, T-maze test), or boldness (Elevated plus maze test). Since exploration and boldness are the most widely studied personality axes in rodents^6^, we decided to focus on these procedures and observe the behaviour laboratory rats exhibit in these tests. We were especially interested in whether the behaviours which are usually recorded in these experiments will be intercorrelated as it is often expected that these procedures measure the same behavioural traits. Moreover, there seems to be a disagreement as to how to interpret the behaviours we actually see during the procedure (this is especially true for the Elevated plus maze test, where various researchers interpret the same behavioural trait quite differently—see Discussion) and how the repeated exposure to the same procedures affects the behaviour of the animals. In addition, these experiments are widely used in behavioural pharmacology (e.g.,^9,41,42^), which means that this study might hold important implications not only for the study of the personality of laboratory animals but also when interpreting the results of various pharmacological studies.

The specific aims of the study were to (i) check if the behaviours exhibited in the experiments meet a basic criterion of personality—repeatability and (ii) examine correlations between the behaviours exhibited in these tests—a behavioural syndrome.

## Methods

### Animals

For the purpose of this study, we obtained 36 male laboratory rats of the Long Evans strain from the breeding facilities within the Czech Academy of Sciences. Experimental animals were divided into two groups, each group consisted of 18 animals from three litters (36 animals from 6 litters in total). The litters were manipulated (female pups and surplus male pups were removed) and we obtained the animals when they were two days old. The adult animals were housed in a box (25 × 25 × 50 cm) in groups consisting of two or three individuals. Throughout the experiment, the rats were kept in standard laboratory conditions—stable temperature (21 °C) and 12-h light–dark-cycle. All animals had ad libitum access to food pellets (Altromin diet 1314) and water.

### General procedure

**Figure 1:**
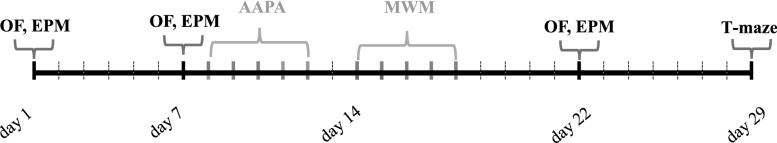
A time axis illustrating the experimental design of this study. Each point on the axis represents one day, bold points represent testing days, dotted points represent days with no experiments. OF—Open field test, EPM—Elevated plus maze test, T-maze—modified T-maze test. Cognitive tasks are shown in grey: AAPA—Active Allothetic Place Avoidance task^43^, MWM—Morris Water Maze task^44^.

During ontogeny, the animals were weighed and tested for motor skills (data are not shown in this study). The experimental testing battery began when the rats reached three months of age. Personality tests featured multiple sessions in order to assess repeatability of behaviour—the Open field test and Elevated plus maze test were repeated three times (in both procedures, the inter-test intervals were five days between the first and second experiment and fourteen days between the second and the third experiment), the T-maze test consisted of four consecutive trials administered on the same day (see Fig. 1). The rats were also tested for aggression in a social interaction test. Analysis of this experiment, however, did not reveal any aggressive behaviour, the results of these experiments are therefore not given here. As a part of a subsequent study, the animals were also tested in cognitive experiments, the results of which will be reported elsewhere. The sequence of the experiments was planned with the main goal of recording and inspecting repeatability estimates from the experiments.

The apparatuses were thoroughly cleaned with Sterilium to eliminate odour cues before the start of each experiment. All experiments were recorded and behavioural parameters scored were selected on the basis of previous studies, e.g.,^7,34,36,45,46^, for the list of parameters recorded and analysed in each procedure see the description of the experiments below. Parameters with minimum frequencies (e.g. freezing) were omitted. All experiments except for the T-maze test were performed without the presence of the experimenter in the room.

All experiments were conducted at the Institute of Physiology of the Czech Academy of Sciences (the department of Neurophysiology of Memory). All animal manipulations were approved by the Ministry of Agriculture committee and done according to the approved project of experiments no. 136/2013. The procedures complied with the Animal Protection Code of Czechia and the appropriate directive of the European Union (2010/63/EC).

### Open field test (OF)

This experiment was invented by Calvin Hall with the aim of studying emotional reactivity in rodents. Individuals that did not move around the arena so much, but defecated a lot, were labeled emotional^46^. Even though the Open field test was originally created for rodents^29^, it benefits from its simplicity and nowadays various species are tested in this paradigm and the Open field test is used for assessing emotionality, activity, and anxiety^8,29,39^.

In our study the apparatus was a brightly illuminated (standard indoor working space illumination—more than 250 lx) white square box (80 × 80 cm) with high walls (80 cm). At the beginning of the experiment, we put the animal into the middle of the apparatus. The open field test was conducted three times (see Fig. 1—experimental design), each trial lasted 10 min.

**Figure 2:**
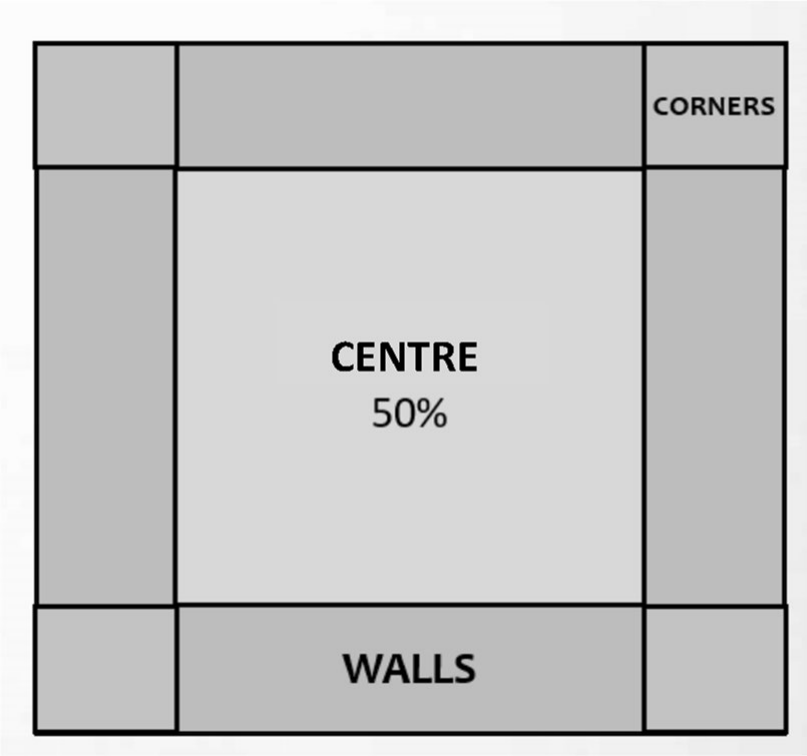
Zones in the Open field test. The centre zone covered 50% of the base of the arena, the rest of the arena was divided into a wall zone and a corner zone.

For the purpose of the statistical analysis, we divided the surface of the apparatus into three zones (see Fig. 2). The centre zone covered 50% of the base, the rest of the arena was divided into a wall and corner zone (all four corners were put together to create one zone called “corner zone”, the same was done for the walls).

Parameters recorded in this experiment were: distance travelled, number of rears, time spent grooming, time spent sitting, and defecation (number of events). Number of rears, grooming and sitting were also analysed for each zone separately as well as number of visits to each zone and time spent there. Video recordings were analysed in EthoVision software platform (Noldus, 10.1 version).

### Elevated plus maze test (EPM)

The Elevated plus maze apparatus is a non-transparent cross-shaped maze, with two open arms (without walls) and two closed arms (with walls), the same arms are situated opposite to each other. This apparatus is used in various areas of research, usually for assessment of exploratory and avoidance behaviours or to study anxiety^8,30,39^. The arms of the maze we used were 50 cm long and had 1 cm high rims on the edges (EPM apparatus used for pharmacological experiments). At the beginning of each trial, the individual animal was placed on an open arm, facing the centre of the maze. The Elevated plus maze was repeated three times (see Fig. 1—scheme of experiments), each trial lasted 10 min.

Parameters recorded in this test: number of entries in the three areas of the maze (open and closed arms, centre zone) and time spent in the three areas of the maze (open and closed arms, centre zone). The recordings were analysed in the Track Analysis program (Biosignal Group, US).

### T-maze test

Variations of this test are used in behavioural testing, cognitive studies and pharmacology, see^47–49^. We used the T-maze to study the animal’s tendency to explore a novel environment. When put into the Y- or T-shaped maze, the animal enters one of the closed arms (to explore or hide in). In the next trial, the animal usually enters the other (unexplored) arm. This behaviour is called spontaneous alternation^33^.

In our variant of this experiment, the rat was placed at the end of an open arm of a T-maze (adapted from the Elevated plus maze apparatus by blocking one open arm) with its head facing the middle of the maze. Its behaviour was recorded until it chose one of the closed arms and went inside, the rat was then quickly retracted from the maze and put into the open arm again (experimental procedure and analysis was adopted similarly to^34^). This procedure was repeated in four subsequent trials during one day.

Parameters recorded in this experiment were: laterality (whether the individual chose the right or the left arm), latency to enter one of the closed arms.

### Analysis and statistics

All parameters were checked for normal distribution and transformed when necessary—count, duration, and latency measures were square root, square root arcsin, and natural logarithm transformed, respectively.

In order to check whether inter-individual differences in behaviour are not solely attributable to differences between litters or the two testing groups (in this study we were not interested in the causes that lead to emergence of inter-individual differences in behaviour, but rather the differences themselves), we performed Linear models with hierarchically organised random effects (lme4 package^50^), using group, litter, and individual identity as random factors. Since most of the variability in behaviour could not be easily attributed to either individual identity, litter, or group (see Table S4 in Supplementary materials), we pooled all animals together for the purpose of this study. In the Results section, we therefore present only the results of pooled analyses.

Firstly, we computed repeatability estimates (both agreement and consistency) for all parameters we used R 3.6.1 (R Core Team 2020^51^) and RStudio (RStudio Team 2020^52^) using the rptR package^53^ (lmm method of repeatability estimation, setting the Gaussian data type and ID as a single random effect).

Secondly, we analysed the inner structure of personality tests (Open field test, Elevated plus maze test) using the Principal Component Analysis in Statistica 9.1 (StatSoft Inc.,2010^54^). For each experiment, we also computed a Parallel analysis in SPSS 20 (IBM Analytics^55^) to assess how many axes or parameters should be used for further analyses in order not to increase the probability of the type I error.

Parallel analysis revealed that only one axis should be used for further analyses. Exploratory analyses revealed original parameters, which expressed the behaviour of the animals extremely well (loading on the first axis of the Principal component analysis was at least 0.79, but mostly over 0.9). Therefore we decided to use the original behavioural variables in subsequent analyses instead of using axes extracted from multivariate analyses. If we were to use the axes derived from the multivariate analyses, the subsequent results would be slightly more accurate but difficult to interpret because we can easily imagine the original behaviour and deduce its meaning. It is, however, increasingly more difficult to do so with synthetic axes representing behaviour. We, therefore, use the primary analyses only to choose the parameters which are then entering the overall multivariate analysis.

After having analysed behaviour in each experiment, we performed an overall analysis of the behaviours measured in the experiments. An elegant way to analyse such data would be a MCMCglmm method^56–60^, however, our sample size did not allow us to use this approach^58^. Instead we performed a Factor Analysis of all personality tests put together so we could investigate relationships between different personality experiments. For this analysis, we used only one parameter from each personality test. The parameters used in the overall analysis were selected according to these three rules: (1) The variable should be repeatable. (2) The variable should represent other parameters from the test—it should comprise as much variability from the first axis of PCA as possible. (3) The variable should be either easily measurable or a standard parameter in the test.

## Results and discussion

### Repeatability of behaviour in laboratory rats

**Figures 3:**
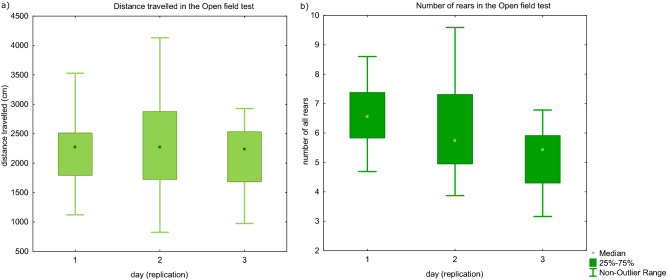
(**a**,**b**) Distance travelled in the Open field test and the number of rears (both supported at the walls and unsupported) in the Open field tests, shown for each replication of the task. Distance travelled did not change across replications, whereas number of rears decreased, probably reflecting habituation to the apparatus.

We detected several parameters with significant repeatability. For the Open field test, the parameters with the highest repeatability were: distance travelled, number of supported rears in the “corner zone” and time spent in active movement (see Fig. 3a and 3b). For the Elevated plus maze test, both time spent in closed and open arms were repeatable, although their repeatability estimates were higher, when changes in time were considered (consistency repeatability). Latency to enter a closed arm in the T-maze test was also repeatable (for all significant repeatability estimates see Table 1).

**Table 1:** Parameters from all personality tests with significant repeatability. Variables with higher repeatability scores than 0.3 are in bold. R_A_ = agreement repeatability, R_C_ = consistency repeatability.

Behavioural test/parameter	R_A_	p	R_C_	P
**Open field test**				
Distance travelled	**0.34**	0.001	**0.33**	0.001
Number of supported rears in the “corner zone”	**0.31**	0.001	**0.32**	0.001
Time spent in active movement	**0.31**	0.002	**0.33**	0.001
Number of unsupported rears	0.18	0.047	**0.33**	0.001
Number of supported rears	0.24	0.012	**0.33**	0.001
Number of visits to the “wall zone”	0.21	0.023	0.26	0.007
Number of unsupported rears in the “corner zone”	0.27	0.006	0.26	0.006
Time spent grooming the “corner zone”	0.22	0.021	0.30	0.002
Time spent sitting in the “corner zone”	0.18	0.049	0.19	0.033
Time spent grooming	0.24	0.013	0.27	0.004
Time spent sitting	0.24	0.012	0.24	0.012
**Elevated plus maze test**				
Time spent in closed arms	0.24	0.012	**0.32**	0.001
Time spent in open arms	0.26	0.007	**0.36**	0.001
**T-maze test**				
Latency to enter one of the closed arms	0.20	0.006	0.23	0.001

### Correlations across tests

From each test, we selected one parameter for further multivariate analyses: distance travelled for the Open field test, time spent in the open arms of the maze for Elevated plus maze test, and latency to enter one of the closed arms for the T-maze test. Distance travelled in the Open field test is usually interpreted as activity, time spent or the number of visits into open arms is usually interpreted as boldness (see Table S1 in Supplementary and its references).

The first factor of a rotated (Varimax normalized) Factor Analysis (F1) explained 19.9% of variability and best correlated with time spent in the open arms in the first (− 0.98) and second (− 0.77) Elevated plus maze test. The second factor (F2) explained 15.5% of variability in the data and correlated with distance travelled in the second Open field test (− 0.93, see Table 2). The T-maze test did not significantly contribute to characterizing and explaining inter-individual differences in our study.

**Table 2:** Factor loadings of variables from personality tests (Open field, Elevated plus maze, and T-maze test). Variables with factor loading 0.7 or higher are in bold.

Factor analysis of personality tests Extraction method: maximum likelihood factors; Varimax normalized rotation		
Variable	Factor 1	Factor 2
Open field test 1_Distance travelled	0.14	− 0.48
Open field test 2_Distance travelled	− 0.21	− **0.93**
Open field test 3_Distance travelled	0.24	− 0.45
Elevated plus maze test 1_Time spent in open arms of the maze	− **0.98**	0.01
Elevated plus maze test 2_Time spent in open arms of the maze	− **0.77**	0.18
Elevated plus maze test 3_Time spent in open arms of the maze	− 0.07	− 0.25
T-maze 1_Latency to enter one of the closed arms	0.50	0.23
T-maze 2_Latency to enter one of the closed arms	0.12	0.01
T-maze 3_Latency to enter one of the closed arms	0.13	0.02
T-maze 4_Latency to enter one of the closed arms	0.17	0.32
Explained variance	19.9%	15.5%

## Discussion

### Consistency of behaviour

The first aim of this study was to look for parameters reflecting personality in the Long Evans strain of laboratory rats. As we have shown in Table 1, we detected several parameters with significant repeatability. However, the consistency of the rats’ behaviour was not very high—the highest repeatability estimate was 0.34 for agreement repeatability (distance travelled in the Open field test) and 0.36 for consistency repeatability (time spent in the open arms of the Elevated plus maze test). Most of the estimates we found were lower than 0.30. According to a meta-analysis concerning the general repeatability of animal behaviour, mean repeatability estimates should be around 0.37^1^.

Despite thorough interbreeding and selection programmes that give rise to laboratory strains (in order to create animals as uniform as possible), studies demonstrate that laboratory animals show behavioural variability (for details on laboratory strains of mice and rats see^8^), it is, however, less pronounced than in wild or wild-derived animals^1,6,7^. The lower repeatability estimates in our data might, therefore, be a explained by the fact that we worked with laboratory animals and we did not manipulate environmental effects (the manipulation was identical for all tested animals) during this study^61–64^. Nevertheless, even though we used a rather uniform set of individuals (belonging to the same laboratory strain) the inter-individual differences in behaviour proved to be significant and not easily negligible.

Moreover, low agreement repeatability and absence of behavioural syndrome could signal habituation to the apparatus when animals are tested repeatedly^65–67^. However, when we calculated consistency repeatability (corrected for systematic changes in behaviour over time), the repeatability estimates did not improve much for most parameters derived from the Open field test. On the other hand, for boldness measured in the Elevated plus maze test, estimates of consistency repeatability were markedly higher than agreement repeatability estimates (see Table 1). Therefore, we can say that we detected a consistent change in boldness across trials in the Elevated plus maze test, probably due to ongoing habituation to the apparatus.

### Behavioural syndrome

Our second aim was to inspect a behavioural syndrome in the Long Evans strain of laboratory rats. However, we found no link between behaviour in the tests we adopted and therefore we did not confirm the existence of a behavioural syndrome using these experimental procedures. Several previous studies have, however, also found significant repeatability of behaviour (personality) without a behavioural syndrome, e.g.,^20,68^.

Even though the Open field test and Elevated plus maze test held the highest predictive value for assessing differences in behaviour (Table 2), these two experimental procedures represented separate orthogonal axes of behaviour in our data. The fact that behaviour in the Open field test is not related to behaviour in the Elevated plus maze test, however, is rather intriguing as these two experiments are sometimes used interchangeably when researchers study exploration or anxiety (see references under the Introduction or Methods sections, or Table S1 in Supplementary materials and its references). In contrast, our results show that these experiments should not be regarded as synonymous, because they seem to test different aspects of behaviour.

The finding that behaviour in the Open field test is not correlated to behaviour in the Elevated plus maze test suggests that interpretation of behavioural parameters from these two experiments should be considered carefully. This finding should be especially interesting for pharmacological studies as they use these procedures quite often and generally consider them interchangeable. In contrast, our results indicate that the behaviour recorded in these two procedures is not equivalent and should be interpreted more cautiously.

Even though behaviour in the T-maze (adapted from EPM test in our case) did not significantly contribute to the explanation of inter-individual behavioural differences of our experimental animals (see Table 1), the result of this experiment is still worth mentioning in light of another study on laboratory rats, where it explained more than 14% of variability in the data^34^. Latency to enter one of the closed arms is also a basis for selection in a selectively bred strain of prematurely aging mice. In this strain of mice, a lower curiosity level (higher latency to enter a closed arm) is positively associated with rapid aging^8^. Results from our experimental setting, however, do not support the notion that motivation to explore (or curiosity) represents a personality trait.

### Systematic changes in behaviour, interpreting the behaviour in the experiments

Another interesting result is that behaviour in these tests changes over time. Subsequent trials of these experiments (especially Open field test and Elevated plus maze test) correlated rather poorly. This finding is in accordance with the results of previous studies^25,31,32^, which also show systematic changes in behaviour in these experiments. These result, however, come from rather older studies and they are often neglected when planning, conducting and interpreting experiments with laboratory rats. Nevertheless, our understanding is that these insights are crucial as overlooking them may cause misinterpretation of the behaviour of the animals.

In the Open field test, a clear pattern has been shown for many species (especially rodents). During the trial, distance travelled in the arena decreases^31^. At first, the animal runs around trying to find a way to escape from the apparatus; towards the end of the trial, the individual ceases to explore the environment and rather engages in grooming, sitting, or rearing^31,69^. This change in behaviour is detectable even across trials^69^. A detailed analysis of repeated exposure to the Open field test, therefore, suggests that the first exposure encompasses both activity and exploration, whereas succeeding exposures reflect activity and habituation to the apparatus^6,25,31,63,70^.

In our data, however, distance travelled did not decrease across trials. Only the number of rears decreased. Therefore, in the second and third trial of the Open field test, the rats moved through the arena, but probably did not try to find a way to escape it. Distance travelled in the Open field test had high loading on the first factor in all three trials of the test (see Table S2 in Supplementary materials) and probably represents activity as has been shown in many other studies (mice^26,41^; rats^27,34^). The slight discrepancy between our results and the previous studies may have been caused by the fact that the animals we tested were not naïve and were accustomed to handling and testing in several other procedures.

Similarly, the first exposure to the Elevated plus maze differs from the succeeding trials^30,32^. Contrary to previous studies (reviewed in^30^), we observed that time spent in the open arms increased across trials, which can be interpreted as the increasing boldness of the tested animals. We also observed systematic changes in explorative behaviours. The distance travelled in the maze and number of entries into the arms (both open and closed) decreased across trials. Therefore, rats showed habituation to the apparatus in this test. The increasing boldness could also be attributed to the fact that our animals were not naïve.

When analysing behaviour in the Elevated plus maze with multivariate methods, the first factor is usually represented by the number of entries into the open arms of the maze or time spent there. This is almost universal among experimental studies and it was also the case for our data (see Table S3 in Supplementary materials). However, the interpretation of this factor and therefore interpretation of the experiment as a whole still seems to be an issue. Various researchers use different interpretations of behaviour recorded in the Elevated plus maze test. Some authors interpret it as “approach/avoidance towards aversive stimuli”^28,71^, others label it as “anxiety”^27,72^, “emotional reactivity”^26^ or “impulsivity”^73^.

## Conclusions

Even though strains of laboratory rats are bred to be homogenous, there are inter-individual differences in behaviour between the animals, which are apparent when the idnividuals are tested in standard behavioural experiments. In this study we confirmed the existence of consistent inter-individual differences (personality) in behaviour in the Long Evans strain of laboratory rats. In several tasks, we found parameters with significant repeatability (ranging from 0.18 to 0.36). This variability is, therefore, rather limited (repeatability estimates were quite low), but still it should not be overlooked when designing and interpreting experiments.

Moreover, repeated exposure to the Elevated plus maze test revealed a consistent systematic change of behaviour among all animals—increasing boldness. Nevertheless, we were not able to confirm a behavioural syndrome among the behaviours measured in the Open field test, Elevated plus maze test, and the T-maze test. However, the absence of a behavioural syndrome in our results is in accordance with several previous studies and suggests that these experimental procedures measure different dimensions of behaviour and are suitable for assessing behaviour corresponding to several personality axes. The results from these procedures should, therefore, be interpreted with caution and these experimental designs should not be used interchangeably.

## Supplementary Information

Supplementary Information.

## Data Availability

Data generated and analysed during this study are available at https://figshare.com/articles/dataset/personality_data_xlsx/17158112. Requests for raw data should be addressed to T.N.

## References

[1] Bell AM, Hankison SJ, Laskowski KL (2009). The repeatability of behaviour: A meta-analysis. Anim. Behav..

[2] Gosling SD (2001). From mice to men: What can we learn about persoanlity from animal research?. Psychol. Bull..

[3] Gosling SD, John OP (1999). Personality dimensions in nonhuman animals: A cross-species review. Curr. Dir. Psychol. Sci..

[4] Réale D, Reader SM, Sol D, McDougall PT, Dingemanse NJ (2007). Integrating animal temperament within ecology and evolution. Biol. Rev..

[5] *Animal Personalities: Behavior, Physiology, and Evolution*. (University of Chicago Press, 2013).

[6] Žampachová B, Landová E, Frynta D (2017). Methods for measuring mammalian personalities: In which animals and how accurately can we quantify it?. Lynx.

[7] Žampachová B, Kaftanová B, Šimánková H, Landová E, Frynta D (2017). Consistent individual differences in standard exploration tasks in the black rat (*Rattus*
*rattus*). J. Comp. Psychol..

[8] Cavigelli SA, Michael KC, Ragan CM, Carere C, Maestripieri D (2013). Behavioral, physiological, and health biases in laboratory rodents: A basis for understanding mechanistic links between human personality and health. Animal Personalities: Behavior, Physiology, and Evolution.

[9] Wang Q (2009). High dose of simvastatin induces hyperlocomotive and anxiolytic-like activities: The association with the up-regulation of NMDA receptor binding in the rat brain. Exp. Neurol..

[10] de Oliveira RMW (2000). Expression of neuronal nitric oxide synthase mRNA in stress-related brain areas after restraint in rats. Neurosci. Lett..

[11] Jessa M, Nazar M, Bidzinski A, Plaznik A (1996). The effects of repeated administration of diazepam, MK-801 and CGP 37849 on rat behavior in two models of anxiety. Eur. Neuropsychopharmacol..

[12] Groothuis TGG, Carere C (2005). Avian personalities: Characterization and epigenesis. Neurosci. Biobehav. Rev..

[13] Digman JM (1990). Personality structure: Emergence of the five-factor model. Annu. Rev. Psychol..

[14] Eysenck HJ (1992). Four ways five factors are not basic. Pers. Individ. Dif..

[15] Costa PTJ, McCrae RR (1992). Four ways five factors are basic. Pers. Individ. Dif..

[16] Réale D (2010). Personality and the emergence of the pace-of-life syndrome concept at the population level. Philos. Trans. R. Soc. B Biol. Sci..

[17] Sih A, Bell A, Johnson JC (2004). Behavioral syndromes: An ecological and evolutionary overview. Trends Ecol. Evol..

[18] Biro PA, Stamps JA (2015). Using repeatability to study physiological and behavioural traits: Ignore time-related change at your peril. Anim. Behav..

[19] Niemelä PT, Vainikka A, Forsman JT, Loukola OJ, Kortet R (2012). How does variation in the environment and individual cognition explain the existence of consistent behavioral differences?. Ecol. Evol..

[20] Šimková, O., Frýdlová, P., Žampachová, B., Frynta, D., & Landová, E. *Development of behavioural profile in the Northern common boa (Boa imperator): Repeatable independent traits or personality? PLoS ONE* **12** (2017). 10.1371/journal.pone.0177911 PMC5443515 28542424

[21] Stamps JA, Groothuis TGG (2010). Developmental perspectives on personality: Implications for ecological and evolutionary studies of individual differences. Philos. Trans. R. Soc. B Biol. Sci..

[22] Lessells CM, Boag PT (1987). Unrepeatable repeatabilities: A common mistake. Auk.

[23] Nakagawa S, Schielzeth H (2010). Repeatability for Gaussian and non-Gaussian data: A practical guide for biologists. Biol. Rev..

[24] Perals D, Griffin AS, Bartomeus I, Sol D (2017). Revisiting the open-field test: What does it really tell us about animal personality?. Anim. Behav..

[25] Denenberg VH (1969). Open field behavior in the rat: What does it mean?. Ann. N. Y. Acad. Sci..

[26] van der Staay, F. J., Schuurman, T., van Reenen, C. G., & Korte, S. M. Emotional reactivity and cognitive performance in aversively motivated tasks: A comparison between four rat strains. *Behav. Brain Funct.* **5** (2009). 10.1186/1744-9081-5-50 PMC2805679 20003525

[27] Ibáñez MI, Ávila C, Ruipérez MA, Moro M, Ortet G (2007). Temperamental traits in mice (I): Factor structure. Pers. Individ. Dif..

[28] Bertoglio LJ, Carobrez AP (2000). Previous maze experience required to increase open arms avoidance in rats submitted to the elevated plus-maze model of anxiety. Behav. Brain Res..

[29] Prut L, Belzung C (2003). The open field as a paradigm to measure the effects of drugs on anxiety-like behaviors: A review. Eur. J. Pharmacol..

[30] Carobrez AP, Bertoglio LJ (2005). Ethological and temporal analyses of anxiety-like behavior: The elevated plus-maze model 20 years on. Neurosci. Biobehav. Rev..

[31] Walsh RN, Cummins RA (1976). The open-field test: A critical review. Psychol. Bull..

[32] Fernandes C, File SE (1996). The influence of open arm ledges and maze experience in the elevated plus-maze. Pharmacol. Biochem. Behav..

[33] Lalonde R (2002). The neurobiological basis of spontaneous alternation. Neurosci. Biobehav. Rev..

[34] Torrejais JCM, Rosa CCM, Boerngen-Lacerda R, Andreatini R (2008). The elevated T-maze as a measure of two types of defensive reactions: A factor analysis. Brain Res. Bull..

[35] Viana MB, Tomaz C, Graeff FG (1994). The elevated T-maze: A new animal model of anxiety and memory. Pharmacol. Biochem. Behavio.

[36] Rodgers RJ, Johnson NJT (1995). Factor analysis of spatiotemporal and ethological measures in the murine elevated plus-maze test of anxiety. Pharmacol. Biochem. Behav..

[37] Rodgers RJ, Cao BJ, Dalvi A, Holmes A (1997). Animal models of anxiety: An ethological perspective. Brazilian J. Med. Biol. Res..

[38] Castro JE (2012). Personality traits in rats predict vulnerability and resilience to developing stress-induced depression-like behaviors, HPA axis hyper-reactivity and brain changes in pERK1/2 activity. Psychoneuroendocrinology.

[39] Krebs R, Linnenbrink M, Guenther A (2019). Validating standardised personality tests under semi-natural conditions in wild house mice (Mus musculus domesticus). Ethology.

[40] Rödel HG, Meyer S (2011). Early development influences ontogeny of personality types in young laboratory rats. Dev. Psychobiol..

[41] Carola V, D’Olimpio F, Brunamonti E, Mangia F, Renzi P (2002). Evaluation of the elevated plus-maze and open-field tests for the assessment of anxiety-related behaviour in inbred mice. Behav. Brain Res..

[42] Ribeiro A, Ferra-de-Paula V, Pinheiro ML, Palermo-Neto J (2009). Dose-response effects of systemic anandamide administration in mice sequentially submitted to the open field and elevated plus-maze tests. Brazilian J. Med. Biol. Res..

[43] Stuchlík, A. *et al.* Place avoidance tasks as tools in the behavioral neuroscience of learning and memory. *Physiol. Res.* **62** (2013). 10.33549/physiolres.932635 24329689

[44] Vorhees CV, Williams MT (2006). Morris water maze: Procedures for assessing spatial and related forms of learning and memory. Nat. Protoc..

[45] D’Hooge, R. & De Deyn, P. P. Applications of the Morris water maze in the study of learning and memory. *Brain Res. Rev. ***36** (2001). 10.1016/s0165-0173(01)00067-4 11516773

[46] Hall CS (1934). Emotional behavior in the rat. I. Defecation and urination as measures of individual differences in emotionality. J. Comp. Psychol..

[47] Deacon RMJ (2006). Appetitive position discrimination in the T-maze. Nat. Protoc..

[48] Locchi F, DallOlio R, Gandolfi O, Rimondini R (2007). Water T-maze, an improved method to assess spatial working memory in rats: Pharmacological validation. Neurosci. Lett..

[49] Zangrossi LIO, Graeff FG (1997). Behavioral validation of the elevated T-maze, a new animal model of anxiety. Brain Res. Bull..

[50] Bates D, Mächler M, Bolker B, Walker S (2015). Fitting linear mixed-effects models using lme4. J. Stat. Softw..

[51] R Core Team. R: A language and environment for statistical computing. R Foundation for Statistical Computing, Vienna, Austria. https://www.R-project.org/ (2020).

[52] RStudio Team. RStudio: Integrated Development for R. RStudio, PBC, Boston, MA. http://www.rstudio.com/ (2020).

[53] Stoffel MA, Nakagawa S, Schielzeth H (2017). rptR: Repeatability estimation and variance decomposition by generalized linear mixed-effects models. Methods Ecol. Evol..

[54] StatSoft Inc. STATISTICA version 9.0., 2009. www.statsoft.com.

[55] IBM Corp. Released 2011. IBM SPSS Statistics for Windows, Version 20.0. IBM Corp.

[56] Hadfield, J. D. 2010 MCMC methods for multi-response generalized linear mixed models: theMCMCglmm R package.J. Stat. Softw.33, 1–22.See http://www.jstatsoft.org/v33/i02/.

[57] Dingemanse NJ (2012). Variation in personality and behavioural plasticity across four populations of the great tit Parus major. J. Anim. Ecol..

[58] Dingemanse NJ, Dochtermann N, Wright J (2010). A method for exploring the structure of behavioural syndromes to allow formal comparison within and between data sets. Anim. Behav..

[59] Mutzel A, Dingemanse NJ, Araya-ajoy YG, Kempenaers B (2013). Parental provisioning behaviour plays a key role in linking personality with reproductive success. Proc. R. Soc. B Biol. Sci..

[60] Dingemanse NJ, Dochtermann NA (2013). Quantifying individual variation in behaviour: Mixed-effect modelling approaches. J. Anim. Ecol..

[61] de Boer SF, van der Vegt BJ, Koolhaas JM (2003). Individual variation in aggression of feral rodent strains: A standard for the genetics of aggression and violence?. Behav. Genet..

[62] Modlinska K, Stryjek R, Pisula W (2015). Food neophobia in wild and laboratory rats (multi-strain comparison). Behav. Processes.

[63] Pisula W, Modlinska K, Chrzanowska A, Stryjek R (2015). Behavioural differences in Brown-Norway and wild-type rats maintained in standard or enriched environment in response to novelty in a familiarised environment. Psychology.

[64] Stryjek R, Modlińska K, Pisula W (2012). Species specific behavioural patterns (digging and swimming) and reaction to novel objects in wild type, Wistar, Sprague-Dawley and Brown Norway rats. PLoS ONE.

[65] Brown GR, Nemes C (2008). The exploratory behaviour of rats in the hole-board apparatus: Is head-dipping a valid measure of neophilia?. Behav. Processes.

[66] Ossenkopp K-P, Sorenson L, Mazmanian DS (1994). Factor analysis of open-field behavior in the rat (*Rattus*
*norvegicus*): Application of the three-way PARAFAC model to a longitudinal data set. Behav. Processes.

[67] Poucet B, Durup M, Thinus-Blanc C (1988). Short-term and long-term habituation of exploration in rats, hamsters and gerbils. Behav. Processes.

[68] Bell AM, Stamps JA (2004). Development of behavioural differences between individuals and populations of sticklebacks, *Gasterosteus*
*aculeatus*. Anim. Behav..

[69] Archer J (1973). Tests for emotionality in rats and mice: A review. Anim. Behav..

[70] Martin JGA, Réale D (2008). Temperament, risk assessment and habituation to novelty in eastern chipmunks, *Tamias*
*striatus*. Anim. Behav..

[71] Ramos A, Berton O, Mormède P, Chaouloff F (1997). A multiple-test study of anxiety-related behaviours in six inbred rat strains. Behav. Brain Res..

[72] Rodgers RJ, Dalvi A (1997). Anxiety, defence and the elevated plus-maze. Neurosci. Biobehav. Rev..

[73] Vevera J (2016). The effect of prolonged simvastatin application on serotonin uptake, membrane microviscosity and behavioral changes in the animal model. Physiol. Behav..

