# Supplementary material for: Inter-individual differences in laboratory rats as revealed by three behavioural tasks
Source: Sci Rep. 2022 Jun 7;12:9361. doi: 10.1038/s41598-022-13288-w (PMC9174278; doi:10.1038/s41598-022-13288-w)
Supplement: Supplementary file 1 — Supplementary Information. [file 41598_2022_13288_MOESM1_ESM.doc]

# Supplementary materials

| **Personality axis** | **Description** | **Experiments** |
| --- | --- | --- |
| **Boldness** - Shyness | reaction to risky situations | Elevated plus maze test ^1–6^ |
|  |  | Novel object test ^1,3,15–24,7,25–34,8,35–40,9–14^ |
|  |  | Startle test (sudden noise or movement ) ^41–44^ |
|  |  | Reaction to handling/ Tonic immobility ^15,21,50–52,28,30,40,45–49^ |
|  |  | Flight initiation distance ^49,53–57^ |
|  |  | Latency to come out of a shelter (or latency to feed) after startle ^13,14,63,27,55,57–62^ |
| **Exploration** - Avoidance | reaction to a new situation  (for example new habitat,  novel food or object) | Open field test ^1,2,46,50,62,64–70,5,71–75,6,15,16,21,29,38,43^ |
|  |  | Hole board test ^76,77^ |
|  |  | Novel environment test ^7,10,78–87,13,88–97,14,98,23,25,26,37,43,70^ |
| **Activity** | general level of activity | The length of a path an animal travels in various experiments ^13,16,58,61,63,73,99–104,17,105,18,28–30,50,55,57^ |
| **Aggressiveness** | tendency to interact agonistically  with conspecifics | Social interaction tests ^16,25,107–111,33,34,47,59,64,75,102,106^ |
|  |  | Competition for food or sexual mate ^13,37,112^ |
| **Sociability** | tendency to amicably associate with conspecifics | Social interaction tests ^2,33,34,58,75^ |

*Table S1: Personality axes, their description and experiments commonly used to study these axes in non-primate animals. For full citations see page 4.*

## Open field test

Principal Component Analyses revealed two factors for each Open field test trial, which explained about 70 % of variability in behaviour in this experiment. For details about factor loading and variability explained by each axis see Table S2.

*Table S2: Results of Principal Component Analyses of the three trials of Open field test (unrotated), each section represents results from one trial.*

| **Variable** | **OF 1**  **PC 1** | **OF 1**  **PC 2** | **OF 2**  **PC 1** | **OF 2**  **PC 2** | **OF 3**  **PC 1** | **OF 3**  **PC 2** |
| --- | --- | --- | --- | --- | --- | --- |
| Distance travelled | **-0.84** | 0.44 | **-0.93** | -0.18 | **-0.94** | -0.11 |
| Number of unsupported rears | **-0.87** | -0.20 | **-0.93** | -0.01 | **-0.81** | -0.17 |
| Number of supported rears | -0.36 | **0.85** | **-0.79** | -0.46 | **-0.71** | -0.23 |
| Time spent grooming | 0.62 | 0.04 | 0.58 | -0.60 | 0.61 | -0.40 |
| Time spent sitting | 0.68 | -0.32 | **0.84** | 0.40 | 0.16 | **0.93** |
| Relative time spent in the “centre” | -0.65 | -0.60 | **-0.76** | 0.32 | **-0.73** | 0.30 |
| Relative time spent in the “corner” | 0.53 | 0.58 | **0.77** | -0.37 | **0.92** | -0.10 |
| **Explained variance** | **44.8 %** | **25.1 %** | **65.1 %** | **14.5 %** | **54.6 %** | **17.5 %** |

Parallel analysis suggested that only one factor or parameter could be used for further analyses According to the rules introduced in the Analysis and statistics section, we chose the parameter “distance travelled” to represent behaviour in the Open field test in further analyses.

Elevated plus maze test

Principal Component Analysis showed that for the first two trials of the Elevated plus maze, only one axis is meaningful, however for the third trial of the test, two axes have eigenvalue higher than 1 (for details of this analysis see Table S3).

*Table S3: Results of Principal Component Analyses of the three trials of Elevated plus maze test (unrotated). Each section represents results from one trial.*

| **Variable** | **EPM 1 PC1** | **EPM 2 PC1** | **EPM 3 PC1** | **EPM 3 PC2** |
| --- | --- | --- | --- | --- |
| Number of entrances into open arms | **-0.85** | **0.81** | **-0.83** | 0.19 |
| Number of entrances into closed arms | **-0.74** | 0.22 | -0.04 | **-0.98** |
| Time spent in open arms | -0.77 | **0.93** | **-0.79** | -0.01 |
| Time spent in closed arms | 0.78 | **-0.94** | **0.77** | 0.25 |
| **Explained variance** | **62.0 %** | **61.3 %** | **47.9 %** | **26.4 %** |

Parallel analysis of the Elevated plus maze test showed that only one parameter should be used for further analyses. Therefore we chose the parameter “time spent in open arms” for further analyses, following the rules introduces in the Analysis and statistics section of this article.

Variability in the data

*Table S4: Variability explained by group, litter and individual identity in personality experiments (linear models with hierarchically organised random effects)*.

|  |  | Variability explained by: | | |
| --- | --- | --- | --- | --- |
| Experiment | Parameter | Group | Litter | Individual identity |
| Open field test | Distance travelled | 24 % | < 1 % | 17 % |
|  | Number of unsupported rears | 25 % | 3 % | 3 % |
|  | Number of supported rears | 21 % | < 1 % | 22 % |
|  | Time spent in active movement | 31 % | < 1 % | 10 % |
|  | Time spent grooming | 29 % | 2 % | 5 % |
|  | Time spent sitting | 3 % | 2 % | 21 % |
| Elevated plus maze test | Time spent in open arms | 13 % | 5 % | 12 % |
|  | Time spent in closed arms | 5 % | 2 % | 2 % |
| T-maze test | Latency to enter an arm | < 1 % | 1 % | 18 % |

1. Castro, J. E. *et al.* Personality traits in rats predict vulnerability and resilience to developing stress-induced depression-like behaviors, HPA axis hyper-reactivity and brain changes in pERK1/2 activity. *Psychoneuroendocrinology* **37**, 1209–1223 (2012).

2. Ramos, A., Berton, O., Mormède, P. & Chaouloff, F. A multiple-test study of anxiety-related behaviours in six inbred rat strains. *Behavioural brain research* **85**, 57–69 (1997).

3. Rangassamy, M. *et al.* Personality modulates proportions of CD4+regulatory and effector T cells in response to socially induced stress in a rodent of wild origin. *Physiol. Behav.* **167**, 255–264 (2016).

4. Rödel, H. G. & Meyer, S. Early development influences ontogeny of personality types in young laboratory rats. *Dev. Psychobiol.* **53**, 601–613 (2011).

5. Kazlauckas, V. *et al.* Behavioral and cognitive profile of mice with high and low exploratory phenotypes. *Behav. Brain Res.* **162**, 272–278 (2005).

6. van der Staay, F. J., Schuurman, T., van Reenen, C. G. & Korte, S. M. Emotional reactivity and cognitive performance in aversively motivated tasks: A comparison between four rat strains. *Behav. Brain Funct.* **5**, (2009).

7. Baugh, A. T. *et al.* Corticosterone responses differ between lines of great tits (Parus major) selected for divergent personalities. *Gen. Comp. Endocrinol.* **175**, 488–494 (2012).

8. Bergvall, U. A., Schäpers, A., Kjellander, P. & Weiss, A. Personality and foraging decisions in fallow deer, Dama dama. *Anim. Behav.* **81**, 101–112 (2011).

9. Blaszczyk, M. B. Boldness towards novel objects predicts predator inspection in wild vervet monkeys. *Anim. Behav.* **123**, 91–100 (2017).

10. Bousquet, C. A. H., Petit, O., Arrivé, M., Robin, J. P. & Sueur, C. Personality tests predict responses to a spatial-learning task in mallards, Anas platyrhynchos. *Anim. Behav.* **110**, 145–154 (2015).

11. Carere, C. & Van Oers, K. Shy and bold great tits (Parus major): Body temperature and breath rate in response to handling stress. *Physiol. Behav.* **82**, 905–912 (2004).

12. Dammhahn, M. & Almeling, L. Is risk taking during foraging a personality trait? A field test for cross-context consistency in boldness. *Anim. Behav.* **84**, 131–1139 (2012).

13. David, M., Auclair, Y. & Cézilly, F. Personality predicts social dominance in female zebra finches, Taeniopygia guttata, in a feeding context. *Anim. Behav.* **81**, 219–224 (2011).

14. Drent, P. J., Van Oers, K. & Van Noordwijk, A. J. Realized heritability of personalities in the great tit (Parus major). *Proc. R. Soc. B Biol. Sci.* **270**, 45–51 (2003).

15. Guenther, A. & Trillmich, F. Within-litter differences in personality and physiology relate to size differences among siblings in cavies. *Physiol. Behav.* **145**, 22–28 (2015).

16. Guenther, A., Finkemeier, M. A. & Trillmich, F. The ontogeny of personality in the wild guinea pig. *Anim. Behav.* **90**, 131–139 (2014).

17. Kluen, E. & Brommer, J. E. Context-specific repeatability of personality traits in a wild bird: A reaction-norm perspective. *Behav. Ecol.* **24**, 650–658 (2013).

18. Kluen, E., Kuhn, S., Kempenaers, B. & Brommer, J. E. A simple cage test captures intrinsic differences in aspects of personality across individuals in a passerine bird. *Anim. Behav.* **84**, 279–287 (2012).

19. Mettke-Hofmann, C., Ebert, C., Schmidt, T., Steiger, S. & Stieb, S. Personality Traits in Resident and Migratory Warbler Species. *Behaviour* **142**, 1357–1375 (2005).

20. Nawroth, C., Prentice, P. M. & McElligott, A. G. Individual personality differences in goats predict their performance in visual learning and non-associative cognitive tasks. *Behav. Processes* **134**, 43–53 (2017).

21. Pittet, F., Coignard, M., Houdelier, C., Richard-Yris, M. A. & Lumineau, S. Effects of maternal experience on fearfulness and maternal behaviour in a precocial bird. *Anim. Behav.* **85**, 797–805 (2013).

22. Schürch, R., Rothenberger, S. & Heg, D. The building-up of social relationships: Behavioural types, social networks and cooperative breeding in a cichlid. *Philos. Trans. R. Soc. B Biol. Sci.* **365**, 4089–4098 (2010).

23. Titulaer, M., van Oers, K. & Naguib, M. Personality affects learning performance in difficult tasks in a sex-dependent way. *Anim. Behav.* **83**, 723–730 (2012).

24. Trompf, L. & Brown, C. Personality affects learning and trade-offs between private and social information in guppies, poecilia reticulata. *Anim. Behav.* **88**, 99–106 (2014).

25. van Horik, J. O., Langley, E. J. G., Whiteside, M. A. & Madden, J. R. Differential participation in cognitive tests is driven by personality, sex, body condition and experience. *Behav. Processes* **134**, 22–30 (2017).

26. van Oers, K., Drent, P. J., de Goede, P. & van Noordwijk, A. J. Realized heritability and repeatability of risk-taking behaviour in relation to avian personalities. *Proc. Biol. Sci.* **271**, 65–73 (2004).

27. Williams, L. J., King, A. J. & Mettke-Hofmann, C. Colourful characters: head colour reflects personality in a social bird, the Gouldian finch, Erythrura gouldiae. *Anim. Behav.* **84**, 159–165 (2012).

28. Wuerz, Y. & Krüger, O. Personality over ontogeny in zebra finches: Long-term repeatable traits but unstable behavioural syndromes. *Front. Zool.* **12**, 59 (2015).

29. Yuen, C. H., Pillay, N., Heinrichs, M., Schoepf, I. & Schradin, C. Personality does not constrain social and behavioural flexibility in African striped mice. *Behav. Ecol. Sociobiol.* **69**, 1237–1249 (2015).

30. Zidar, J. *et al.* A comparison of animal personality and coping styles in the red junglefowl. *Anim. Behav.* **130**, 209–220 (2017).

31. Bannier, F., Tebbich, S. & Taborsky, B. Early experience affects learning performance and neophobia in a cooperatively breeding cichlid. *Ethology* **123**, 712–723 (2017).

32. Bebus, S., Small, T. W., Jones, B. C., Elderbrock, E. K. & Schoech, S. J. Associative learning is inversely related to reversal learning and varies with nestling corticosterone exposure. *Anim. Behav.* **111**, 251–260 (2016).

33. Brust, V. & Guenther, A. Domestication effects on behavioural traits and learning performance : comparing wild cavies to guinea pigs. *Anim. Cogn.* **18**, 99–109 (2015).

34. Brust, V. & Guenther, A. Stability of the guinea pigs personality – cognition – linkage over time. *Behav. Processes* **134**, 4–11 (2017).

35. Gibelli, J. & Dubois, F. Does personality affect the ability of individuals to track and respond to changing conditions ? *Behav. Ecol.* **28**, 101–107 (2017).

36. Kareklas, K., Elwood, R. W. & Holland, R. A. Personality effects on spatial learning : Comparisons between visual conditions in a weakly electric fish. *Ethology* **123**, 551–559 (2017).

37. Lermite, F., Peneaux, C. & Griffin, A. S. Personality and problem-solving in common mynas (Acridotheres tristis). *Behav. Processes* **134**, 87–94 (2017).

38. Mazza, V., Eccard, J. A., Zaccaroni, M., Jacob, J. & Dammhahn, M. The fast and the flexible: cognitive style drives individual variation in cognition in a small mammal. *Anim. Behav.* **137**, 119–132 (2018).

39. Nawroth, C., Prentice, P. M. & Mcelligott, A. G. Individual Personality Differences in Goats Predict Their Performance in Visual Learning and Non- Associative Cognitive Tasks. *Behav. Processes* **134**, 43–53 (2016).

40. Zidar, J. *et al.* The relationship between learning speed and personality is age- and task-dependent in red junglefowl. *Behav. Ecol. Sociobiol.* **72**, 168 (2018).

41. Osborn, A. & Briffa, M. Does repeatable behaviour in the laboratory represent behaviour under natural conditions? A formal comparison in sea anemones. *Anim. Behav.* **123**, 197–206 (2017).

42. Pruitt, J. N., Grinsted, L. & Settepani, V. Linking levels of personality: Personalities of the ‘average’ and ‘most extreme’ group members predict colony-level personality. *Anim. Behav.* **86**, 391–399 (2013).

43. Schuster, A. C., Zimmermann, U., Hauer, C. & Foerster, K. A behavioural syndrome , but less evidence for a relationship with cognitive traits in a spatial orientation context. 1–14 (2017). doi:10.1186/s12983-017-0204-2

44. Stanley, C. R., Mettke-hofmann, C. & Preziosi, R. F. Personality in the cockroach Diploptera punctata : Evidence for stability across developmental stages despite age effects on boldness. 1–24 (2017).

45. Brommer, J. E. & Kluen, E. Exploring the genetics of nestling personality traits in a wild passerine bird: Testing the phenotypic gambit. *Ecol. Evol.* **2**, 3032–3044 (2012).

46. Careau, V. *et al.* Energy expenditure and personality in wild chipmunks. *Behav. Ecol. Sociobiol.* **69**, 653–661 (2015).

47. D’Eath, R. B. & Burn, C. C. Individual Differences in Behaviour : A Test of ’ Coping Style ’ Does Not Predict Resident- Intruder Aggressiveness in Pigs. *Behaviour* **139**, 1175–1194 (2002).

48. Kluen, E., Siitari, H. & Brommer, J. E. Testing for between individual correlations of personality and physiological traits in a wild bird. *Behav. Ecol. Sociobiol.* **68**, 205–213 (2014).

49. Petelle, M. B., McCoy, D. E., Alejandro, V., Martin, J. G. A. & Blumstein, D. T. Development of boldness and docility in yellow-bellied marmots. *Anim. Behav.* **86**, 1147–1154 (2013).

50. Šimková, O., Frýdlová, P., Žampachová, B., Frynta, D. & Landová, E. *Development of behavioural profile in the Northern common boa (Boa imperator): Repeatable independent traits or personality?* *PLoS ONE* **12**, (2017).

51. Brust, V., Wuerz, Y. & Krüger, O. Behavioural Flexibility and Personality in Zebra Finches. *Ethology* **119**, 559–569 (2013).

52. de Haas, E. N., Lee, C., Hernandez, C. E., Naguib, M. & Rodenburg, T. B. Individual differences in personality in laying hens are related to learning a colour cue association. *Behav. Processes* **134**, 37–42 (2017).

53. Carter, A. J., Goldizen, A. W. & Tromp, S. A. Agamas exhibit behavioral syndromes: Bolder males bask and feed more but may suffer higher predation. *Behav. Ecol.* **21**, 655–661 (2010).

54. Carter, A. J., Heinsohn, R., Goldizen, A. W. & Biro, P. A. Boldness, trappability and sampling bias in wild lizards. *Anim. Behav.* **83**, 1051–1058 (2012).

55. Niemelä, P. T., Lattenkamp, E. Z. & Dingemanse, N. J. Personality-related survival and sampling bias in wild cricket nymphs. *Behav. Ecol.* **26**, 936–946 (2015).

56. Sol, D. *et al.* Risk-taking behavior , urbanization and the pace of life in birds. (2018).

57. Wilson, A. D. M. & Krause, J. Personality and metamorphosis: Is behavioral variation consistent across ontogenetic niche shifts? *Behav. Ecol.* **23**, 1316–1323 (2012).

58. Michelangeli, M., Wong, B. B. M. & Chapple, D. G. It’s a trap: Sampling bias due to animal personality is not always inevitable. *Behav. Ecol.* **27**, 62–67 (2016).

59. Riechert, S. E. & Hedrick, A. V. A test for correlations among fitness-linked behavioural traits in the spider Agelenopsis aperta(Araneae, Agelenidae). *Anim. Behav.* **46**, 669–675 (1993).

60. Stein, L. R., Trapp, R. M. & Bell, A. M. Do reproduction and parenting influence personality traits? Insights from threespine stickleback. *Anim. Behav.* **112**, 247–254 (2016).

61. Urszán, J. T. *et al.* Experience during development triggers between-­ individual variation in behavioural plasticity. *J. Anim. Ecol.* **87**, 1264–1273 (2018).

62. Yuen, C. H., Schoepf, I., Schradin, C. & Pillay, N. Boldness : are open fi eld and startle tests measuring the same personality trait ? *Anim. Behav.* **128**, 143–151 (2017).

63. Urszán, J. T. *et al.* No personality without experience? A test on Rana dalmatina tadpoles. *Ecol. Evol.* **5**, 5847–5856 (2015).

64. Bengston, S. E., Pruitt, J. N. & Riechert, S. E. Differences in environmental enrichment generate contrasting behavioural syndromes in a basal spider lineage. *Anim. Behav.* **93**, 105–110 (2014).

65. Gyuris, E., Feró, O. & Barta, Z. Personality traits across ontogeny in firebugs, Pyrrhocoris apterus. *Anim. Behav.* **84**, 103–109 (2012).

66. Herde, A. & Eccard, J. A. Consistency in boldness, activity and exploration at different stages of life. *BMC Ecol.* **13**, (2013).

67. Lantová, P., Zub, K., Koskela, E., Šíchová, K. & Borowski, Z. Is there a linkage between metabolism and personality in small mammals? The root vole (Microtus oeconomus) example. *Physiol. Behav.* **104**, 378–383 (2011).

68. Lantová, P., Brixová, L. & Lanta, V. Urine marking in male common voles: Does behavioural activity matter? *Behav. Processes* **90**, 174–179 (2012).

69. Perals, D., Griffin, A. S., Bartomeus, I. & Sol, D. Revisiting the open-field test: what does it really tell us about animal personality? *Anim. Behav.* **123**, 69–79 (2017).

70. Schuster, A. C., Carl, T. & Foerster, K. Repeatability and consistency of individual behaviour in juvenile and adult Eurasian harvest mice. 1–14 (2017). doi:10.1007/s00114-017-1430-3

71. Šíchová, K., Koskela, E., Mappes, T., Lantová, P. & Boratyński, Z. On personality, energy metabolism and mtDNA introgression in bankvoles. *Anim. Behav.* **92**, 229–237 (2014).

72. Zipser, B., Kaiser, S. & Sachser, N. Dimensions of Animal Personalities in Guinea Pigs. *Ethology* **119**, 970–982 (2013).

73. DePasquale, C., Wagner, T., Acgard, G. A., Ferguson, B. & Braithwaite, V. A. Learning rate and temperament in a high predation risk environment. *Oecologia* **176**, 661–667 (2014).

74. D’Ettore, P. *et al.* Individual differences in exploratory activity relate to cognitive judgement bias in carpenter ants. *Behav. Processes* **134**, 63–69 (2017).

75. Udino, E., Perez, M., Carere, C. & D’Etorre, P. Active explorers show low learning performance in a social insect. *Curr. Zool.* **63**, 555–560 (2017).

76. Ray, J. & Hansen, S. Temperamental development in the rat: The first year. *Dev. Psychobiol.* **47**, 136–144 (2005).

77. Žampachová, B., Kaftanová, B., Šimánková, H., Landová, E. & Frynta, D. Consistent individual differences in standard exploration tasks in the black rat (Rattus rattus). *J. Comp. Psychol.* **131**, 150–162 (2017).

78. Aplin, L. M. *et al.* Individual personalities predict social behaviour in wild networks of great tits (Parus major). *Ecol. Lett.* **16**, 1365–1372 (2013).

79. Aplin, L. M., Farine, D. R., Mann, R. P. & Sheldon, B. C. Individual-level personality influences social foraging and collective behaviour in wild birds. *Proc. R. Soc. B Biol. Sci.* **281**, 20141016 (2014).

80. Arvidsson, L. K., Adriaensen, F., van Dongen, S., De Stobbeleere, N. & Matthysen, E. Exploration behaviour in a different light: testing cross-context consistency of a common personality trait. *Anim. Behav.* **123**, 151–158 (2017).

81. Bajer, K. *et al.* European green lizard (Lacerta viridis) personalities: Linking behavioural types to ecologically relevant traits at different ontogenetic stages. *Behav. Processes* **111**, 67–74 (2015).

82. Baugh, A. T., van Oers, K., Naguib, M. & Hau, M. Initial reactivity and magnitude of the acute stress response associated with personality in wild great tits (Parus major). *Gen. Comp. Endocrinol.* **189**, 96–104 (2013).

83. Bouwhuis, S., Quinn, J. L., Sheldon, B. C. & Verhulst, S. Personality and basal metabolic rate in a wild bird population. *Oikos* **123**, 56–62 (2014).

84. Cole, E. F. & Quinn, J. L. Personality and problem-solving performance explain competitive ability in the wild. *Proc. R. Soc. B Biol. Sci.* **279**, 1168–1175 (2012).

85. Dingemanse, N. J., Both, C., Drent, P. J., Van Oers, K. & Van Noordwijk, A. J. Repeatability and heritability of exploratory behaviour in great tits from the wild. *Anim. Behav.* **64**, 929–938 (2002).

86. Dingemanse, N. J. *et al.* Variation in personality and behavioural plasticity across four populations of the great tit Parus major. *J. Anim. Ecol.* **81**, 116–126 (2012).

87. Guillette, L. M., Reddon, A. R., Hurd, P. L. & Sturdy, C. B. Exploration of a novel space is associated with individual differences in learning speed in black-capped chickadees, Poecile atricapillus. *Behav. Processes* **82**, 265–270 (2009).

88. Jacobs, C. G. C. *et al.* Personality-dependent response to field playback in great tits: Slow explorers can be strong responders. *Anim. Behav.* **90**, 65–71 (2014).

89. Johnson, K. V. A. *et al.* Male great tits assort by personality during the breeding season. *Anim. Behav.* **128**, 21–32 (2017).

90. Naguib, M., Van Rooij, E. P., Snijders, L. & Van Oers, K. To sing or not to sing: seasonal changes in singing vary with personality in wild great tits. *Behav. Ecol.* **27**, 932–938 (2016).

91. Nicolaus, M., Tinbergen, J. M., Ubels, R., Both, C. & Dingemanse, N. J. Density fluctuations represent a key process maintaining personality variation in a wild passerine bird. *Ecol. Lett.* **19**, 478–486 (2016).

92. van Overveld, T. & Matthysen, E. Personality and Information Gathering in Free-Ranging Great Tits. *PLoS One* **8**, (2013).

93. Snijders, L. *et al.* Social networking in territorial great tits: Slow explorers have the least central social network positions. *Anim. Behav.* **98**, 95–102 (2014).

94. Guillette, L. M., Reddon, A. R., Hoeschele, M. & Sturdy, C. B. Sometimes slower is better: slow-exploring birds are more sensitive to changes in a vocal discrimination task. *Proc. R. Soc. B Biol. Sci.* **278**, 767–773 (2011).

95. Guillette, L. M., Hahn, A. H., Hoeschele, M., Przyslupski, A.-M. & Sturdy, C. B. Individual differences in learning speed, performance accuracy and exploratory behaviour in black-capped chickadees. *Anim. Cogn.* **18**, 165–178 (2015).

96. Dougherty, L. R. & Guillette, L. M. Linking personality and cognition: A meta-analysis. *Philos. Trans. R. Soc. B Biol. Sci.* **373**, (2018).

97. Guillette, L. M., Baron, D. M., Sturdy, C. B. & Spetch, M. L. Fast- and slow-exploring pigeons differ in how they use previously learned rules. *Behav. Processes* **134**, 54–62 (2017).

98. Moiron, M., Mathot, K. J. & Dingemanse, N. J. A multi-level approach to quantify speed-accuracy trade-offs in great tits (Parus major). *Behav. Ecol.* **27**, 1539–1546 (2016).

99. Brust, V., Wuerz, Y. & Krüger, O. Behavioural Flexibility and Personality in Zebra Finches. *Ethology* **119**, 559–569 (2013).

100. Mazzamuto, M. V. *et al.* Rodents in the arena: a critical evaluation of methods measuring personality traits. *Ethol. Ecol. Evol.* **31**, 38–58 (2019).

101. Mitchell, D. J. & Biro, P. A. Is behavioural plasticity consistent across different environmental gradients and through time? *Proc. R. Soc. B Biol. Sci.* **284**, 20170893 (2017).

102. Merten, S. Von, Zwolak, R. & Rychlik, L. Social personality : a more social shrew species exhibits stronger differences in personality types. **127**, 125–134 (2017).

103. Wilson, A. D. M. & Godin, J. G. J. Boldness and behavioral syndromes in the bluegill sunfish, Lepomis macrochirus. *Behav. Ecol.* **20**, 231–237 (2009).

104. Bensky, M. K., Paitz, R., Pereira, L. & Bell, A. M. Testing the predictions of coping styles theory in threespined sticklebacks. *Behav. Processes* **136**, 1–10 (2017).

105. Lalot, M., Ung, D., Péron, F., D’Ettore, P. & Bovet, D. You know what? I’m happy. Cognitive bias is not related to personality but is induced by pair-housing in canaries (Serinus canaria). *Behav. Processes* **134**, 70–77 (2017).

106. Araya-Ajoy, Y. G. & Dingemanse, N. J. Repeatability, heritability, and age-dependence of seasonal plasticity in aggressiveness in a wild passerine bird. *J. Anim. Ecol.* **86**, 227–238 (2017).

107. Bell, A. M. & Stamps, J. A. Development of behavioural differences between individuals and populations of sticklebacks, Gasterosteus aculeatus. *Anim. Behav.* **68**, 1339–1348 (2004).

108. Coppens, C. M., De Boer, S. F., Steimer, T. & Koolhaas, J. M. Correlated behavioral traits in rats of the roman selection lines. *Behav. Genet.* **43**, 220–226 (2013).

109. Coppens, C. M., de Boer, S. F., Steimer, T. & Koolhaas, J. M. Impulsivity and aggressive behavior in Roman high and low avoidance rats: Baseline differences and adolescent social stress induced changes. *Physiol. Behav.* **105**, 1156–1160 (2012).

110. Gracceva, G., Koolhaas, J. M. & Groothius, T. G. G. Does the Early Social Environment Affect Structure and Consistency of Personality in Wild-Type Male Rats? *Dev. Psychobiol.* **53**, 614–623 (2011).

111. Matzel, L. D., Kolata, S., Light, K. & Sauce, B. The tendency for social submission predicts superior cognitive performance in previously isolated male mice. *Behav. Processes* **134**, 12–21 (2017).

112. Pruitt, J. N., Riechert, S. E. & Jones, T. C. Behavioural syndromes and their fitness consequences in a socially polymorphic spider, Anelosimus studiosus. *Anim. Behav.* **76**, 871–879 (2008).
